# Ectopic pregnancy: a single-center experience over ten years

**DOI:** 10.1186/s12958-021-00761-w

**Published:** 2021-06-01

**Authors:** Ammar Al Naimi, Pablo Moore, Dörthe Brüggmann, Lisa Krysa, Frank Louwen, Franz Bahlmann

**Affiliations:** 1 Department of Obstetrics and Gynecology, University Hospital, Goethe University Frankfurt am Main, Frankfurt, Hessen Germany; 2 Department of Obstetrics and Gynecology, Buergerhospital - Dr. Senckenbergische Stiftung, Nibelungenallee 37-41, D-60318 Frankfurt am Main, Hessen Germany; 3 High Risk Pregnancy Unit, University Hospital of Puerto Montt, Puerto Montt, Chile

**Keywords:** Ectopic pregnancy, Heterotopic pregnancy, Dizygotic ectopic, β-HCG

## Abstract

**Purpose:**

The aim of this study was to investigate characteristics associated with ectopic pregnancy (EP) that could be utilized for predicting morbidity or mortality.

**Methods:**

This was a retrospective analysis of pregnancy-related records from a tertiary center over a period of ten years. Data on age, gravidity, parity, EP risk, amenorrhea duration, abdominal pain presence and location, β-human chorionic gonadotropin (β-HCG) level, ultrasound findings, therapeutic intervention, exact EP implantation site and length of hospital stay (LOS) were obtained from the database. The LOS was used as a proxy for morbidity and was tested for an association with all variables. All statistical analyses were conducted with Stata® (ver. 16.1, Texas, USA).

**Results:**

The incidence of EP in a cohort of 30,247 pregnancies over a ten-year period was 1.05%. Patients presented with lower abdominal pain in 87.9% of cases, and the likelihood of experiencing pain was tenfold higher if fluid was detectable in the pouch of Douglas. Only 5.1% of patients had a detectable embryonic heartbeat, and 18.15% had one or more risk factors for EP. While most EPs were tubal, 2% were ovarian. The LOS was 1.9 days, and laparoscopic intervention was the main management procedure. The cohort included one genetically proven dizygotic heterotopic pregnancy (incidence, 3.3 × 10^− 5^) that was diagnosed in the 7th gestational week. The only association found was between the β-HCG level and LOS, with a linear regression β coefficient of 0.01 and a *P*-value of 0.04.

**Conclusion:**

EP is a relatively common condition affecting approximately 1% of all pregnancies. β-HCG correlates with EP-related morbidity, but the overall morbidity rate of EP is low regardless of the implantation site. Laparoscopic surgery is an effective therapeutic procedure that is safe for managing EP, even in cases of heterotopic pregnancy.

## Synopsis

Ectopic pregnancy is a relatively common condition with an overall low morbidity rate. Even in cases of heterotopic pregnancy, laparoscopic surgery is a safe and effective therapeutic option.

## Introduction

Ectopic pregnancy (EP) refers to the implantation of a gestational sac outside the uterine cavity, and its incidence is approximately 1% in Western women of reproductive age [1, 2] The classical locations for EP implantation are cervical, interstitial, isthmic, ampullary, fimbrial and ovarian [3]. Furthermore, tubal EPs are the most commonly diagnosed, accounting for approximately 94% of EPs [4]. The increasing rate of cesarean section has led to the identification of the hysterotomy scar as an additional site for EP implantation, and cesarean scar EP ranges from less than 1 to 6% of all EPs [5, 6]. Aside from cesarean section, other known risk factors for EP include previous EP, smoking, pelvic inflammatory disease, use of a contraceptive intrauterine devices (IUD), and use of assisted reproductive techniques (ARTs) [7]. Heterotopic pregnancy (HP) is a rare condition in which an EP simultaneously accompanies an intrauterine pregnancy [8]. When EP is not properly diagnosed, it is considered a serious gynecological emergency due to the possibility of tubal rupture and intraperitoneal bleeding. Therefore, complicated EPs are associated with significant morbidity and even mortality [9]. The traditional management of EP involves surgical intervention with salpingostomy. Nevertheless, timely diagnosis allows for conservative medical management with methotrexate (MTX), which is most effective in the early stages of EP [10, 11]. The presentation of up to 10% of EP patients is acute due to tubal rupture and hemodynamic instability leading to morbidity exacerbation [12]. These patients might face additional reductions in quality of life due to a compromised reproductive capacity. Therefore, several authors have emphasized the importance of identifying factors of morbidity factors to develop countermeasures [13]. The aim of this study was to investigate EP-associated characteristics that could be utilized for predicting morbidity or mortality.

## Methods

This was a retrospective analysis of pregnancy-related records from a tertiary center over a period of ten years. The database was screened for patients diagnosed with EP, and the files of these patients were reviewed for information of interest. The variables collected for the study included demographic data, signs and symptoms, clinical examinations, management strategies, and outcomes. These were age, gravidity, parity, history of EP, IUD use, ART use, amenorrhea duration, abdominal pain presence and location, β-human chorionic gonadotropin (β-HCG) level, ultrasound findings (e.g., an embryonic heartbeat and fluid in the pouch of Douglas), type of therapeutic intervention, exact EP implantation site and length of hospital stay (LOS).

We utilized the LOS, which is a continuous outcome measured in days, as a proxy for morbidity, with a longer period of hospitalization indicating greater morbidity. The association between our collected variables and the outcome was tested to identify potential predictors of morbidity. Analysis was based on linear regression, chi-square tables, logistic regression and multivariate regression, with *P* < 0.05 indicating significance. All statistical analyses were conducted with Stata® (ver. 16.1, Texas, USA).

## Results

Our records showed a total of 30,247 pregnancies and 319 EPs within the period of ten years from 2008 to 2018. The incidence of EP in our cohort was 1.05%, and it predominantly occurred on the right side. Patients presented with lower abdominal pain in 87.9% of cases, and 18.15% had one or more risk factors for EP. While ultrasound showed free fluid in the pouch of Douglas in the majority of patients, only 5.1% had a detectable embryonic heartbeat. There was a significant association between pain and fluid in the pouch of Douglas (*P* < 0.001), and the odds ratio of experiencing pain when fluid was present was tenfold. Almost all of cases of EP were tubal, and only 2% were ovarian. Laparoscopic intervention was the main management method, and the mean LOS was 1.9 days. The database contained one HP case; this patient presented with lower abdominal pain and was diagnosed in the 7th gestational week. In this case, the viable intrauterine pregnancy survived a laparoscopic salpingostomy and progressed until labor in the 36th gestational week. The baby was a healthy girl, while genetic analysis of the EP showed a 46, XY karyotype. The main findings from our cohort are summarized in Table 1.

**Table 1:** Summary of the distribution of the demographic characteristics, signs, symptoms, and management of the study cohort

Variable	Proportion/mean ± standard deviation	Median	Interquartile range
Age (years)	33.16 ± 5.04	33.6	20.1–45.6
Gravidity	2.52 ± 1.43	2	1–7
Parity	0.78 ± 0.89	1	0–3
Gestational age (weeks)	6.41 ± 1.55	6	3–9
Risk for EP	18.15%	–	
β-HCG (mIU/mL)	5509 ± 4131	1930	44–74,754
Laterality	right: 54.5%	–	
	left: 45.4%		
Location	ampullary: 43.8%	–	
	fimbrial: 37.4%		
	isthmic: 16.8%		
	ovarian: 2%		
Abdominal pain	87.9%	–	
Fluid in the pouch of Douglas	86.9%	–	
Detectable heartbeat	5.1%	–	
Intervention	salpingostomy: 97.3%	–	
	salpingectomy: 2%		
	methotrexate: 0.7%		
Length of stay (days)	1.92 ± 1.4	2	1–7

Linear regression was applied to test the association between the numerous study variables and the outcome of interest, which was the LOS in days. The only statistically significant association was found between the LOS and the β-HCG level measured in IU/mL, with a β coefficient of 0.01 and *P*-value of 0.04. The results of the linear regression for all study variables are shown in Table 2.

**Table 2:** Results of linear regression of various variables with the duration of hospitalization in days showing the regression β coefficient, *P*-value, and 95% confidence interval (CI)

	β Coefficient	P-value	95% Confidence interval
β-HCG (IU/mL)	0.01	0.04	0.0006 - 0.022
Gestational age (weeks)	0.06	0.39	−0.08 - 0.2
Location	−0.01	0.96	−0.22 - 0.21
Detectable heartbeat	0.02	0.95	−0.68 - 0.73
Fluid in the pouch of Douglas	0.35	0.15	−0.16 - 0.82
Abdominal pain	0.28	0.26	−0.21 - 0.76
Intervention method	0.05	0.89	−0.69 - 0.8
Gravidity	0.01	0.85	−0.13 - 0.16
Laterality	−0.13	0.41	−0.44 - 0.18
Age (years)	0.02	0.25	−0.01 - 0.05

Building a regression model for β-HCG as a predictor of morbidity and adjusting for all variables, including age, gestational age, location, ascites, pain, and management method, resulted in a β coefficient of 0.01 and a *P*-value of 0.2.

## Discussion

The incidence of EP in this large cohort was approximately 1%, which mirrors the quoted incidence in the literature and might suggest that our cohort is population-representative [2]. Even though our cohort included no cases of cesarean scar EP, the literature shows an increase in its incidence. This trend might not be solely caused by the increase in cesarean births but rather due to increased awareness of the condition improving the diagnosis. A similar development was observed in the incidence of ovarian EP, which continuously increased up to 1997 and remained steady afterward [14].

The site of EP implantation affects the severity of complications. Interstitial EP, for example, is associated with a high likelihood of hemorrhage and thus increased morbidity [3]. Interestingly, the site of implantation in our cohort showed no association with the LOS. The fact that almost all our EP cases were tubal and that complicated cases, such as cervical or cesarean scar EP, were missing could have skewed our results toward the null.

Two-thirds of EPs are observed in multigravidas; therefore, higher gravidity is considered a risk factor for EP [15]. The mean gravidity of 2.52 in the study cohort confirms this statement, but neither gravidity nor parity affected morbidity.

**Fig. 1:**
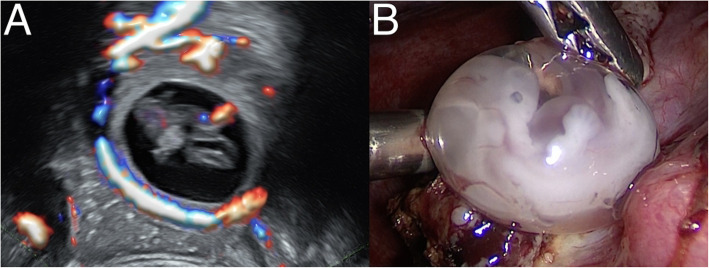
**A** Sonographic appearance of an ectopic pregnancy showing the ‘ring of fire’ sign and an embryonic cardiac signal. **B** Laparoscopically extracted embryonic sac

It is crucial to exclude EP in women of reproductive age presenting with amenorrhea combined with either bleeding or abdominal pain. While β-HCG confirms the presence of a pregnancy, it alone cannot provide evidence of an EP. Ultrasound is an essential examination for the diagnosis of EP, and while embryonic cardiac activity is rarely seen, free fluid in the pouch of Douglas, an empty uterine cavity and adnexal masses are common signs of EP. Approximately 85% of EPs are diagnosed prior to tubal rupture [2]. Doppler ultrasound can improve the sensitivity to approximately 90% by displaying the classic sign of the ‘ring of fire’ around the adnexal mass that is pathognomonic for EP, as shown in Fig. 1 [16].

Left undiagnosed, EP ends up in rupture, hemorrhage, and hypovolemic shock, with a pregnancy-related mortality rate of 9% [17]. There were no cases of mortality in our cohort, which is expected in a Western first-world setting. Our cohort showed no association between sonographic findings and morbidity, but delayed diagnosis is a known cause of morbidity. Missed diagnosis leads to tubal rupture, hypovolemic shock, blood transfusions and salpingectomy, which are usually associated with high morbidity [4]. Our cohort was diagnosed on average in the seventh gestational week. This early diagnosis combined with the short LOS could explain the lack of an association between the gestational age and LOS. Moreover, the positive association between the β-HCG level and LOS is an indicator of increased morbidity with increasing gestational age. Recent work has shown that factors affecting patients’ access to healthcare, such as the COVID-19 pandemic, delay the timely diagnosis of EP and increase morbidity and mortality by increasing the rate of tubal rupture and bleeding [18].

**Fig. 2:**
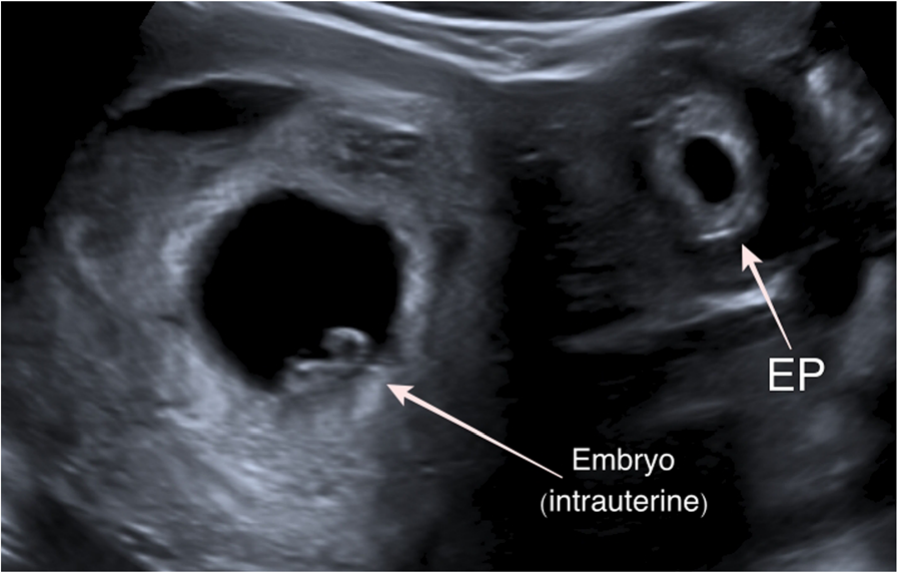
Sonographic diagnosis of a heterotopic pregnancy showing a typical three-layered ectopic adnexal mass (EP) alongside a viable intrauterine embryo

This database is especially interesting for including a rare case of HP, which is basically a twin pregnancy where one embryo reaches the uterine cavity and implants normally, whereas the other is ectopic. Therefore, HP is assumed to result primarily from a dizygotic pregnancy [8]. We believe this case to be the first published case of genetically tested HP with proven dizygosity. The incidence of spontaneous HP was reported to be 1 in 30,000 pregnancies, but the increased use of ARTs might have led to an increase in the incidence of up to 1 in 3889 [19, 20]. The incidence of HP in our cohort is in accordance with the former report. The presence of an intact intrauterine pregnancy significantly affects the diagnostic power of ultrasound in identifying an EP. Almost half of the EPs in cases of HP are missed during the initial screening and are only diagnosed after repeated sonographic examinations [21]. Without the presence of embryonic structures in cases of HP and due to the low incidence, the adnexal mass can be mistaken for a corpus luteum cyst. Therefore, careful examination of every adnexal mass resembling an EP, as shown in Fig. 2, is required, even if an intact intrauterine pregnancy is evident.

Surgical intervention with either laparoscopic salpingostomy or salpingectomy is the usual strategy applied for the management of EP regardless, of whether the EP is complicated with rupture [4]. Almost all of our cases were managed surgically, and only 2% of them required salpingectomy. Medical management is a cost-effective therapeutic alternative, and a single dose of MTX has a median success rate of 84% [22]. Medical treatment is more favorable than surgical options when well-defined selection criteria are met. These recommended criteria for MTX therapy, including an asymptomatic presentation, a small intact gestational sac, a low β-HCG level and no embryonic cardiac activity, are often associated with early EP diagnosis [23]. The rate of MTX use in our cohort is unusually low, especially compared to multicenter data from the United Kingdom, where 30% of EPs are successfully managed with MTX [24]. This difference could be attributed to the fact that our data represent data from a single center. Moreover, the eventual need for second-line interventions, post-MTX abstinence, and enthusiastic surgeons with confidence in performing laparoscopy are factors that lead to the limited use of MTX. Some practitioners perform curettage with laparoscopy, but this practice should be avoided to decrease the incidence of iatrogenic uterine scarring and spare intrauterine pregnancies in cases of HP. Moreover, surgery is the only available option for treating HP, and some authors recommend salpingectomy in these cases due to the increased risk of bleeding [25]. The case of HP in this cohort was successfully managed with salpingostomy without adverse outcomes. Therefore, the recommendation of salpingectomy deserves objective scrutiny.

This study exhibits weaknesses inherent to the retrospective design. The lack of a power analysis could be responsible for our inability to show an association between most of the study variables and the outcome due to the study being underpowered. The structure of the database shapes the analysis and therefore introduces information bias. Hemoglobin, for example, could have been utilized for assessing bleeding and morbidity, but this information was not available. Morbidity and LOS demonstrate a direct correlation; therefore, the LOS has been utilized as a measure of morbidity in the context of complicated EP [26]. Nevertheless, a hospital discharge database might not be able to detect cases of complicated EP with increased morbidity due to incorrect coding practices [27].

In conclusion, EP is a relatively common condition affecting approximately 1% of all pregnancies. β-HCG correlates with EP-related morbidity, but the overall morbidity rate of EP is low regardless of the implantation site. Laparoscopic surgery is an effective therapeutic procedure that is safe for managing EP, even in cases of HP.

## Data Availability

Not applicable.

## References

[1] Chow WH, Daling JR, Cates W, Greenberg RS (1987). Epidemiology of ectopic pregnancy. Epidemiol Rev.

[2] Job-Spira N, Fernandez H, Bouyer J, Pouly JL, Germain E, Coste J (1999). Ruptured tubal ectopic pregnancy: risk factors and reproductive outcome: results of a population-based study in France. Am J Obstet Gynecol.

[3] Bouyer J, Coste J, Fernandez H, Pouly JL, Job-Spira N (2002). Sites of ectopic pregnancy: a 10 year population-based study of 1800 cases. Hum Reprod.

[4] Suresh A, Devasia JM, Adla B, Balachandran A, YVS (2019). A study on ectopic pregnancies in a tertiary care centre. J Evid Based Med Healthcare.

[5] Seow KM, Huang LW, Lin YH, Lin MY, Tsai YL, Hwang JL (2004). Cesarean scar pregnancy: issues in management. Ultrasound Obstet Gynecol.

[6] Kumari V, Kumar H, Datta MR. The Importance of Ectopic Mindedness: Scar Ectopic Pregnancy, a Diagnostic Dilemma. Cureus. 2021;13(2):e13089. 10.7759/cureus.13089. 10.7759/cureus.13089 PMC7933601 33728112

[7] Job-Spira N, Collet P, Coste J, Brémond A, Laumon B (1993). Risk factors for ectopic pregnancy. Results of a case control study in the Rhone-Alpes region. Contracept Fertil Sex.

[8] Wallach EE, Tal J, Haddad S, Gordon N, Timor-Tritsch I (1996). Heterotopic pregnancy after ovulation induction and assisted reproductive technologies: a literature review from 1971 to 1993. Fertil Steril.

[9] Alkatout I, Honemeyer U, Strauss A, Tinelli A, Malvasi A, Jonat W, Mettler L, Schollmeyer T (2013). Clinical diagnosis and treatment of ectopic pregnancy. Obstet Gynecol Surv.

[10] Lipscomb GH (2007). Medical therapy for ectopic pregnancy. Semin Reprod Med.

[11] The Practice Committee of the American Society for Reproductive Medicine. Medical treatment of ectopic pregnancy: a committee opinion. Fertil Steril. 2013;100(3):638–44. 10.1016/j.fertnstert.2013.06.013 23849842

[12] Odejinmi F, Sangrithi M, Olowu O (2011). Operative laparoscopy as the mainstay method in management of hemodynamically unstable patients with ectopic pregnancy. J Minim Invasive Gynecol.

[13] McGurk L, Oliver R, Odejinmi F (2019). Severe morbidity with ectopic pregnancy is associated with late presentation. J Obstet Gynaecol.

[14] Raziel A, Golan A, Pansky M, Ron-El R, Bukovsky I, Caspi E (1990). Ovarian pregnancy: a report of twenty cases in one institution. Am J Obstet Gynecol.

[15] Ayaz A, Emam S, Farooq MU (2013). Clinical course of ectopic pregnancy: a single-center experience. J Hum Reprod Sci.

[16] Singhal M, Ahuja CK, Saxena AK, Dhaliwal L, Khandelwal N (2010). Sonographic appearance of heterotopic pregnancy with ruptured ectopic tubal pregnancy. J Clin Ultrasound.

[17] Control CfD, Prevention (1995). Ectopic pregnancy--United States, 1990-1992. MMWR Morb Mortal Wkly Rep.

[18] Dvash S, Cuckle H, Smorgick N, Vaknin Z, Padoa A, Maymon R (2021). Increase rate of ruptured tubal ectopic pregnancy during the COVID-19 pandemic. Eur J Obstetrics Gynecol Reprod Biol..

[19] DeVoe RW, Pratt JH (1948). Simultaneous intrauterine and extrauterine pregnancy. Am J Obstet Gynecol.

[20] Bello GV, Schonholz D, Moshirpur J, Jeng D-Y, Berkowitz RL (1986). Combined pregnancy: the Mount Sinai experience. Obstet Gynecol Surv.

[21] Yu Y, Xu W, Xie Z, Huang Q, Li S (2014). Management and outcome of 25 heterotopic pregnancies in Zhejiang, China. Eur J Obstetrics Gynecol Reprod Biol.

[22] Stovall TG, Ling FW (1993). Single-dose methotrexate: an expanded clinical trial. Am J Obstet Gynecol.

[23] Obstetricians ACo, Gynecologists. ACOG Practice Bulletin No. 94: medical management of ectopic pregnancy. Obstet Gynecol 2008;111(6):1479–1485, DOI: 10.1097/AOG.0b013e31817d201e. 10.1097/AOG.0b013e31817d201e 18515537

[24] Taheri M, Bharathan R, Subramaniam A, Kelly T (2014). A United Kingdom national survey of trends in ectopic pregnancy management. J Obstet Gynaecol.

[25] Harzif AK, Hyaswicaksono P, Kurniawan RH, Wiweko B (2021). Heterotopic pregnancy: diagnosis and pitfall in ultrasonography. Gynecol Minim Invasive Ther.

[26] San Lazaro Campillo IS, Meaney S, O’Donoghue K, Corcoran P (2018). Ectopic pregnancy hospitalisations: A national population-based study of rates, management and outcomes. Eur J Obstet Gynecol Reprod Biol.

[27] Fermaut M, Fauconnier A, Brossard A, Razafimamonjy J, Fritel X, Serfaty A (2019). Detection of complicated ectopic pregnancies in the hospital discharge database: a validation study. PLoS One.

